# Increased genital mucosal cytokines in Canadian women associate with higher antigen-presenting cells, inflammatory metabolites, epithelial barrier disruption, and the depletion of *L. crispatus*

**DOI:** 10.1186/s40168-023-01594-y

**Published:** 2023-07-25

**Authors:** Christina Farr Zuend, Alana Lamont, Laura Noel-Romas, Samantha Knodel, Kenzie Birse, Kateryna Kratzer, Peter McQueen, Michelle Perner, Hossaena Ayele, Sarah Mutch, Alicia R. Berard, John J. Schellenberg, Faruk Senturk, Stuart McCorrister, Garrett Westmacott, Fran Mulhall, Bonnie Sandberg, Adelicia Yu, Margaret Burnett, Vanessa Poliquin, Adam D. Burgener

**Affiliations:** 1 Center for Global Health and Diseases, Department of Pathology, Case Western Reserve University, Cleveland, USA; 2 Department of Medical Microbiology and Infectious Diseases, University of Manitoba, Winnipeg, Canada; 3 Department of Obstetrics, Gynecology & Reproductive Sciences, University of Manitoba, Winnipeg, Canada; 4 JC Wilt Infectious Disease Research Centre, National Microbiology Laboratory, Public Health Agency of Canada, Winnipeg, Canada; 5 Health Sciences Centre, Winnipeg, Canada; 6 Unit of Infectious Diseases, Department of Medicine Solna, Center for Molecular Medicine, Karolinska Institute, Karolinska University Hospital, Stockholm, Sweden

**Keywords:** Inflammation, Antigen-presenting cells, Microbiome, Metaproteome, Metabolome

## Abstract

**Background:**

Cervicovaginal inflammation has been linked to negative reproductive health outcomes including the acquisition of HIV, other sexually transmitted infections, and cervical carcinogenesis. While changes to the vaginal microbiome have been linked to genital inflammation, the molecular relationships between the functional components of the microbiome with cervical immunology in the reproductive tract are understudied, limiting our understanding of mucosal biology that may be important for reproductive health.

**Results:**

In this study, we used a multi’-omics approach to profile cervicovaginal samples collected from 43 Canadian women to characterize host, immune, functional microbiome, and metabolome features of cervicovaginal inflammation. We demonstrate that inflammation is associated with lower amounts of *L. crispatus* and higher levels of cervical antigen-presenting cells (APCs). Proteomic analysis showed an upregulation of pathways related to neutrophil degranulation, complement, and leukocyte migration, with lower levels of cornified envelope and cell-cell adherens junctions. Functional microbiome analysis showed reductions in carbohydrate metabolism and lactic acid, with increases in xanthine and other metabolites. Bayesian network analysis linked *L. crispatus* with glycolytic and nucleotide metabolism, succinate and xanthine, and epithelial proteins SCEL and IVL as major molecular features associated with pro-inflammatory cytokines and increased APCs.

**Conclusions:**

This study identified key molecular and immunological relationships with cervicovaginal inflammation, including higher APCs, bacterial metabolism, and proteome alterations that underlie inflammation. As APCs are involved in HIV transmission, parturition, and cervical cancer progression, further studies are needed to explore the interactions between these cells, bacterial metabolism, mucosal immunity, and their relationship to reproductive health.

Video Abstract

**Supplementary Information:**

The online version contains supplementary material available at 10.1186/s40168-023-01594-y.

## Introduction

Cervicovaginal inflammation, typically defined as elevated levels of pro-inflammatory cytokines, has been linked to increased rates of acquisition for sexually transmitted infections (STIs) including human immunodeficiency virus (HIV), diminished efficacy of topical pre-exposure prophylaxis (PrEP) for HIV prevention, cervical carcinogenesis, preterm birth, and long-term reproductive health complications such as pelvic inflammatory disease and tubal infertility [1–15]. Sustained inflammation can lead to increases in proteolytic enzymes, cause damage to the cervicovaginal epithelial barrier, and lead to increased immune cell infiltration or activation, which are thought to be potential mechanisms that increase the risk of HIV acquisition and lead to poor prognosis in cervical cancer [4, 16–20]. While many factors can modulate cervicovaginal inflammation including STIs and hormonal birth control, the vaginal microbiome is a major contributor [1, 21–23]. However, the relationships between the microbiome, mucosal immunity, and cervicovaginal inflammation are not well understood.

Epithelial and immune cells present in the cervicovaginal mucosa monitor the local microenvironment and initiate the production of cytokines and chemokines such as interleukin (IL)-1β, IL-6, and IL-8, and the recruitment or activation of leukocytes [24, 25]. The cervicovaginal mucosa is populated with a dynamic population of innate and adaptive immune cells including neutrophils, macrophages and monocytes, natural killer (NK) cells, T cells, and B cells [24–26]. The cervicovaginal immune system typically exists in homeostasis with the local microbiota [6, 27]. Typically the optimal vaginal microbiome is dominated by species of *Lactobacillus*, which are associated with low levels of inflammation, including low levels of pro-inflammatory cytokines such as IL-1, IL-8, tumor necrosis factor alpha (TNFα), and interferon gamma (IFNγ) [1, 23, 27–29]. Vaginal microbial dysbiosis, defined by a loss of *Lactobacillus* and an overgrowth of obligate and facultative anaerobes such as *Gardnerella*, *Atopobium*, or *Mobiluncus* [30–34], can also affect the mucosal microenvironment and have been associated with increases in pro-inflammatory cytokines, including IL-1β, IL-6, IL-8, and TNFα, resulting in recruitment, maturation, and activation of antigen-presenting cells (APCs), neutrophils, and CD4+ T cells [3, 4, 27–29, 35–49]. Indeed, the composition of the microbiome has been shown to be strongly associated with genital inflammation [3]. The modulation of inflammation is thought to be, in part, driven by metabolites derived from dysbiotic bacterial communities, such as short-chain fatty acids, amino acids, lipids, carbohydrates, and xenobiotics [6, 8, 50–54]. Conversely, lactate, produced by vaginal *Lactobacillus*, elicits anti-inflammatory effects from cervicovaginal epithelial cells, including suppression of IL-6, IL-8, and TNFα production [55, 56]. While these studies have provided insight into how the vaginal microbiome may modulate specific aspects of inflammation, the relationships between pro-inflammatory cytokines, resident immune cells, metabolites, and epithelial barrier function have been understudied limiting our understanding of potential mechanisms contributing to infectious disease susceptibility and other negative reproductive health outcomes.

In this study, we used a multi’-omics approach to profile cervicovaginal samples collected from Canadian women to characterize host, microbiome, and metabolome features of cervicovaginal inflammation. Using an integrated Bayesian network approach, we identify novel interactions linking vaginal mucosal inflammation with differences in the metabolome, microbiome, and APCs which may be important for mucosal health in the female genital tract.

## Materials and methods

### Study population

Forty-three women were recruited from gynecology clinics in Winnipeg, Canada, as part of the Vaginal Mucosal Systems (VMS) study. Enrollment criteria included age over 18 years, HIV negative, and no current or suspected pregnancy. All women provided written informed consent and the study was approved by the Institutional Review Board at the University of Manitoba.

### Data and sample collection

Socio-demographic, obstetric, and gynecological data was collected by structured questionnaire. Physicians performed pelvic examinations and collected an APTIMA multi-test swab, 2 mid-vaginal swabs (Starplex Scientific Inc., Etobicoke, ON, rayon swabs, stored dry until processing), cervicovaginal lavage (CVL), 2 endocervical cytobrush samples, and touched a pH strip to vaginal wall. The APTIMA swab was sent to CADHAM provincial laboratory in Winnipeg, Canada, for chlamydia and gonorrhea testing. All other samples were immediately stored on ice and transported to the lab within 3 h of collection.

### Cervicovaginal lavage

CVL was collected by bathing the cervical os with 10mL of sterile phosphate-buffered saline (PBS). CVL was aliquoted and stored at −80°C until use.

### Cytokine array

25μL CVL was analyzed using EMD Millipore’s MILLIPLEX MAP 30-plex Human Cytokine/Chemokine Magnetic Bead Panel following the manufacturer’s procedures. Samples were randomized across the assay plate, and 25 samples were randomly selected to be analyzed in duplicate. Cytokines and chemokines that were below the limit of detection in ≥40% of samples were removed from analysis. Cytokines below the limit of detection were imputed using half of the minimum detectable concentrations. A curated list of 10 inflammatory cytokines (interferon α2 (IFN-α2), interleukin (IL)-1α, IL-1β, IL-6, IL-8, IFN-γ inducible protein 10 (IP-10), monocyte chemoattractant protein 1 (MCP-1), macrophage inflammatory protein (MIP)-1α, MIP-1β, and Regulated upon Activation, Normally T cell Expressed and Secreted (RANTES)) [2, 4, 5] were used to define inflammation within this study.

### Endocervical cytobrush

Cytobrushes were vortexed for 45 s in PBS to dislodge cells, washed twice in fresh PBS, and filtered twice prior to staining with an optimized antibody cocktail: CD19-APC (clone HIB19), CD56-PE-Cy7 (clone B159), CD4-APC-H7 (clone RPA-T4), CD3-v500 (clone UCHT1), CD8-BV605 (clone SK1), CD49d-PE-Cy5 (clone 9F10), CD14-PerCP-Cy5.5 (clone MφP9), CD16-Alexa700 (clone 3G8), CD15-BUV395 (clone HI98), Fixable viability stain 570 (all antibodies obtained from BD Biosciences). Samples were fixed in 1% paraformaldehyde prior to acquisition on an LSR II (BD Biosciences). Human peripheral blood mononuclear cells were used for staining controls and fluorescence minus one (FMO) controls. Data was analyzed using FlowJo v10 (Treestar).

### Diagnosis of bacterial vaginosis

A wet mount microscopy slide was created using 30μL vaginal swab eluate and observed at 400× magnification for the presence of Clue cells [57]. A Whiff test was performed by mixing 30μL eluate with 30μL of 10% KOH, with detection of a strong amine odor indicating a positive result [57]. A pH strip that had been touched to the vaginal wall was used to measure vaginal pH. Discharge color and consistency were assessed by the study physician during pelvic exam. A participant was diagnosed with bacterial vaginosis (BV) if they had 3 out of 4 Amsel criteria present.

### Sample preparation for proteomics analysis

CVL supernatants were prepared for mass spectrometry as previously described [58]. Briefly, BCA assay (Novagen) was used to quantify protein and then equal volumes were used. Samples were denatured with 8M urea, reduced using diothiothreitol, alkylated using iodoacetamide, and digested into peptides using trypsin. CVL pellets were collected by centrifuging 2mL CVL at 21,000×g for 5 min at 4°C. Pellets were resuspended in 350μL lysis buffer (2% SDS, 0.1 M dithiothreitol, 0.5 M HEPES), then 50μL 0.1 mm glass beads were added. Samples were vortexed, heated at 95°C for 5 min, then vortexed for 3 min. Samples were centrifuged at 3000 rpm for 3 min to pellet beads and cells, and the supernatant was transferred to a fresh tube on ice. This was repeated 3 times. Protein quantification was determined using 2-D Quant assay (GE Healthcare, NJ, USA), and protein was denatured using 8M urea, alkylated using iodoacetamide, then treated with benzonase solution (250 U/μL benzonase, 50 mM MgCl_2_, 50 mM HEPES), before being digested by trypsin. Peptides were cleaned of salts and detergents by reverse-phase liquid chromatography (LC) using the step-function gradient. Peptides were then quantified using a LavaPep Fluorescent Peptide and Protein Quantification Kit (Gel Company, CA, USA) following the manufacturer’s protocol. One microgram of peptide per sample was re-suspended in 2% acetonitrile with 0.1% formic acid submitted for nanoflow-liquid chromatography-tandem mass spectrometry (LC-MS/MS) analysis.

### LC-MS/MS setup for proteomics analysis

Each sample was separately analyzed using a nano-flow Easy nLC 1000 connected in-line to a Q-Exactive Plus mass spectrometer with a nanoelectrospray ion source at 2 kV (Thermo Fisher Scientific, San Jose, CA, USA). The peptide samples were loaded (1 μg) onto a C18-reversed phase Easy Spray column (50 cm long, 75-μm inner diameter, 2-μm particles (Thermo Fisher Scientific, San Jose, CA, USA)) with 100% buffer A (2% acetonitrile, 0.1% formic acid) for a total volume of 10 μl, and then separated on the same column. Peptides were eluted using a linear gradient of 5–22% buffer B (98% acetonitrile, 0.1% formic acid) over 100 min, 22–32% buffer B for 15 min, 32–90% buffer B for 5 min, and a wash at 90% B for 10 min at a constant flow rate of 200 nl/min. Total LC/MS/MS run-time was about 180 min, including the loading, linear gradient, column wash, and the equilibration.

### MS data acquisition

Dynamically choosing the top 15 abundant precursor ions from each survey scan for isolation in the quadrupole (1.4 m/z isolation width) and fragmentation by HCD (28% normalized collision energy). The survey scans were acquired in the orbitrap over m/z 375–1500 with a target resolution of 70,000 at m/z 200, and the subsequent fragment ion scans were also acquired in the orbitrap with a resolution of 17,500 at m/z 200. The lower threshold for selecting a precursor ion for fragmentation was 1.9e4. Dynamic exclusion was enabled using a list size of 500 features, a m/z tolerance of 15 ppm, a repeat count of 1, and an exclusion duration of 20 s.

### Human proteome data analysis

Raw MS spectra were imported into Progenesis QI software (v2.0, Nonlinear Dynamics), aligned, and filtered as described previously [21, 59]. Filtered peptides were annotated using Mascot Daemon (v.2.4.0, Matrix Science) and searched against the SwissProt/UniProtKB (2015) human database, with a decoy database included to determine the rate of false discovery. Protein identifications were confirmed using Scaffold software (v4.8.3, Proteome Software) with confidence thresholds set at ≤1% FDR protein at the protein level, ≤0.1% FDR at the peptide level, and had ≥2 unique peptides identified per protein. Normalized relative abundances of each protein within each sample were obtained from Progenesis QI. Only proteins that had an average covariance of <25% (925 proteins) as determined through measurements of a standard reference sample run at 10 sample intervals (*n*=7) were used in downstream analysis to exclude proteins with higher technical measurement variability. Pathway analysis of host proteins was performed using ConsensusPathDB-human [60–63].

### Microbial proteome data analysis

Protein database searches were conducted against an in-house vaginal metaproteome (VMP) database [64] using Mascot (v.2.4.0, Matrix Science) and included human proteins from the SwissProt/UniprotKB database to limit potential homologous identifications. Search results were then imported into Scaffold software to validate these protein identifications, filtered using the following criteria: <0.1%FDR for peptide identification, $\le$ 1% FDR for protein identification, and at least two unique peptides identified per protein, and protein spectral counts were normalized to total protein detected. Microbial taxa abundance was estimated by taking the sum of normalized total spectral counts from Scaffold for all proteins associated with each genus. Scaffold accession reports containing homologous protein information were used to identify proteins that matched to more than one genus, which were binned into an “undistinguishable” category.

### Functional microbiome analysis

Non-homologous bacterial proteins identified in each participant were annotated for known biological functions using the KEGG ontology database with GhostKOALA (v2.2, Kyoto University Bioinformatics Center) [65]. Proteins were binned to the pathway level; a total of 33 pathways were analyzed for interactions with inflammation to preserve experimental power.

### Sample preparation for metabolomics analysis

Metabolomics procedures were adapted from Srinivasan et al. [52]. Metabolites were extracted from CVL using a 1:4 ratio of CVL to methanol, followed by vortexing and centrifugation at 16,000×g for 30 min at 4°C. The supernatants were transferred to new tubes and dried using vacuum centrifugation. Metabolite samples were resuspended in 200μL of sample buffer (30% Buffer A: 70% Buffer B containing 53.1 μM ^13^C_5_-^15^N_1_-glutamic acid and 58.2 μM ^13^C_2_-succinic acid. Buffer A = 5 mM ammonium acetate in 0.1% acetic acid, Buffer B = 0.1% acetic acid in acetonitrile), then vortexed for 15 min and centrifuged at 16,000×g for 30 min to remove particulate matter. A standard sample containing a mixture of 10 known metabolites, along with a mix sample consisting of extracted metabolites from a mixture of all samples, was injected periodically throughout each batch run for QC and monitoring LC-MS/MS conditions.

### Targeted LC-MS/MS metabolomics analysis

Metabolites were separated using an Agilent 1200 binary pump HPLC system equipped with a ZIC-cHILIC column (150 × 2.1 mm, 3.0 μm particle size) (Millipore). The flow rate was set to 200 μL/min and the column temperature was kept at 4°C. The separation gradient was set as follows: 0–3 min: 70% B, 3–7.5 min: 70–30% B, 7.5–13.5 min: 30% B, 13.5–16.5 min: 30–70% B, 16.5–27 min: 70% B. Eight microliters of each sample was injected for simultaneous positive and negative mode analysis on a Fusion Lumos Tribrid mass spectrometer. Targeted quantification of metabolites was accomplished in parallel reaction monitoring (PRM) mode, targeting 121 different metabolites (66 in positive mode, 55 in negative mode). Source conditions for positive mode were set as follows: Spray Voltage: 3500 V, Sheath Gas: 3 (Arbitrary Units), Aux Gas: 1.2 (Arbitrary Units), Ion Transfer Tube Temp: 275°C. Source conditions for negative mode are the same as for positive except the Spray Voltage is set to 2100 V. Mass spectrometry data is acquired by alternating MS1 and MS2 scans in both positive and negative mode. MS1 scans are collected with the following parameters (the same for positive and negative unless listed otherwise): Scan range: 55–280 m/z (positive), 65–500 (negative), Orbitrap resolution: 30,000, RF Lens (%): 30, AGC Target: 4.0e5, Max Injection Time: 50 ms. MS2 scans are collected with the following parameters (the same for positive and negative mode unless listed otherwise): Isolation Window: 1.6 m/z, HCD Collision Energy: 30%, Stepped Collision Energy: +/− 10%, Orbitrap Resolution: 15,000, Maximum Injection Time 22 ms. For MS2 scans, the mass range was set from 50 m/z to the mass of the targeted metabolite + 10 m/z. Raw files obtained are subsequently uploaded into Skyline for metabolite peak integration and quantification using a custom method. A zero replacement strategy was used to impute values for metabolites below the limit of detection, and metabolites that were below the limit of detection in ≥40% of samples were removed from the analysis.

### Statistical analysis

Statistical analysis and data visualization was performed with GraphPad Prism 8 (v.8.3.1 (332)) or R (version 4.1.3) with plugins “NMF” (version 0.24.0), “RColorBrewer” (version 1.1-2), “dendextend” (version 1.16.0), and “ggplot2” (version 3.3.5). Demographic and gynecological data was analyzed using chi-square or Kruskal-Wallis with Dunn’s multiple comparison test. Multi-‘omics data was analyzed using Kruskal-Wallis with Benjamini-Hochberg correction for multiple comparisons.

### Bayesian Network analysis

Random forest models were created for each multi-‘omic dataset (vaginal metaproteome taxa, vaginal metaproteome functions, vaginal metaproteome proteins, metabolome, host proteome, cytokine, and flow cytometry) using the 3-level inflammation variable as the classifier. All continuous data was discretized using above/below median prior to network creation. Datasets were first screened using Random Forest models [66] in R (randomForest, version 4.7-1.1) to assess the top 5 most important variables based on Gini importance, which were selected as features for the network. The inflammation variable was also included in the network, resulting in 36 features chosen as nodes. Four participants were removed from this analysis as they did not have complete information across datasets. Discretized data from the 36 selected features across 39 participants were used as input for the Bayesian Network, which was constructed using the BNlearn package in R (version 4.8.1). To avoid getting stuck at a possible local maximum, 1000 networks were bootstrapped and averaged for 1000 random restarts, resulting in 1,000,000 total networks. The final consensus network was calculated using the average.network function in BNlearn [67]. For each network created, the structure of the network was learned using the Hill-Climbing algorithm with the likelihood-equivalence Bayesian Dirichlet (BDe) scoring method. The optimal imagery sample size was calculated with the alpha.star function. As the network is intended to be a hypothesis-generating tool, the presence of an edge describes a relationship between two nodes without directionality. The final graph was visualized in Gephi using the Fruchterman Reingold layout. The nodes are colored by data type and size indicates degree of the node.

## Results

### Study participants

Forty-three women were recruited from gynecology clinics in Winnipeg, Canada, as part of the Vaginal Mucosal Systems (VMS) study. Most participants self-reported as Caucasian (37, 86%). Sixteen participants (37%) self-reported having at least 1 vaginal symptom in the past month, including discomfort, itchiness, discharge, or bleeding. Ten participants (23%) had cervical ectopy. Twenty-three (53%) reported intercourse in the past 30 days, with 2 (5%) reporting anal intercourse, and 9 (21%) reporting using condoms. Use of hormonal contraceptives was low, with 12 participants (30%) reporting use, primarily of intrauterine devices (IUDs). Twelve (28%) had a history of any sexually transmitted infection, with 1 participant testing positive for chlamydia at the study visit.

**Fig. 1:**
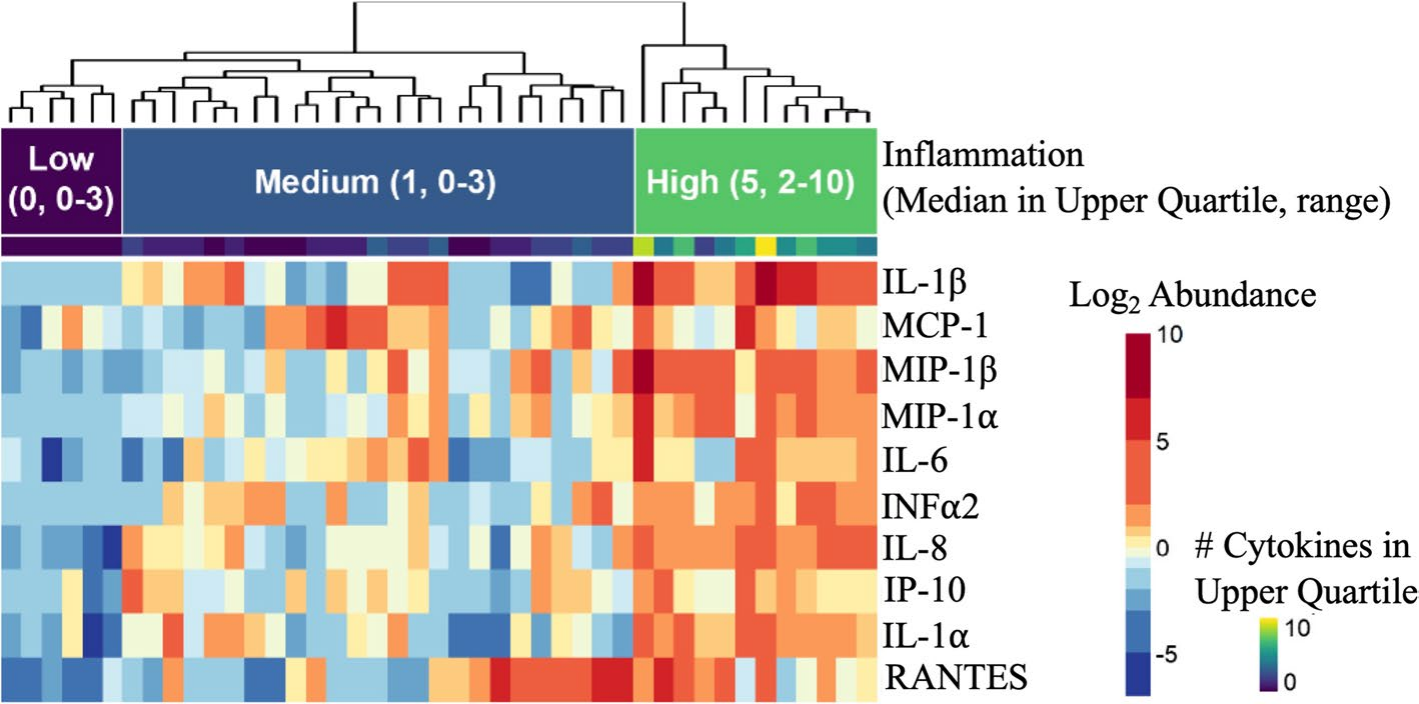
Heatmap showing clustering of inflammation groups based on cytokine levels in cervicovaginal fluid of study participants. Inflammatory cytokines were quantified in CVL using Luminex technology. Unsupervised hierarchical clustering was used to group participants by low, medium, or high levels of inflammation. *N*=43

We classified participants into low, medium, or high genital inflammation groups (Table 1) based on the concentrations of 10 inflammatory cytokines in CVL that have been previously linked with increased acquisition of HIV (IFN-α2, IL-1α, IL-1β, IL-6, IL-8, IP-10, MCP-1, MIP-1α, MIP-1β, and RANTES) [2, 4, 5] using unsupervised hierarchical clustering (Fig. 1). Women in the low inflammation group (*n*=6) had a median of 0 (range 0–3) inflammatory cytokines in the upper quartile, while women in the medium inflammation group (*n*=25) had a median of 1 (range 0–3) inflammatory cytokines in the upper quartile, and women in the high inflammation group (*n*=12) had a median of 5 (range 2–10) inflammatory cytokines in the upper quartile. Vaginal pH was significantly lower (Kruskal-Wallis *p*=0.034, H statistic=6.75, Degrees of freedom=2) in the low inflammation group compared to the medium (p.adj=0.064) and high (p.adj=0.037) inflammation groups. There were no significant differences in age, Nugent score, bacterial vaginosis (BV) diagnosed using Amsel criteria, or *Lactobacillus* dominance of the microbiota (defined as >50% microbial proteins from *Lactobacillus* species) between groups (Table 1). IL-1β was the only cytokine that difference based on *Lactobacillus* dominance of the microbiome (*p*=0.021) and was increased in non-*Lactobacillus* dominant (nLD) participants.

**Table 1:** Overview of participant characteristics

**Variable category**	**Low inflammation (*n*=6)**	**Medium inflammation (*n*=25)**	**High Inflammation (*n*=12)**	***p* value**
Ethnicity (Caucasian) no., %	5 (83%)	22 (88%)	9 (75%)	0.598^^^
Age (median ± SD; range)	25 ± 11.2 (19–46)	37 ± 12.1 (21–64)	42.5 ± 24.04 (23–88)	0.171^χ^
Vaginal pH (median ± SD; range)	4.25 ± 0.24 (4.0–4.7)	4.7 ± 0.44 (4.0–5.5)	4.8 ± 0.46 (4.0–5.8)	0.034^χ^
BV+ (Amsel criteria), no., %	0 (0%)	4 (16%)	4 (33%)	0.248^
Vaginal symptoms in the past month, any (no., %)^a^	4 (67%)	9 (36%)	3 (25%)	0.222^^^
Ectopy (no., %)	0 (0%)	7 (28%)	3 (25%)	0.473^^^
Any intercourse past 30 days	4 (67%)	15 (60%)	4 (42%)	0.631^^^
Condom use (no., %)	0 (0%)	6 (24%)	3 (25%)	0.598^^^
Any birth control use	1 (17%) Oral—1	10 (40%) Oral—2 Ring—2 IUD—6	2 (17%) Oral—1 IUD—1	0.328^^^
Any STI, ever (no., %)	0 (0%)	10 (40%)	2 (17%)	0.117^^^
*Lactobacillus* dominant microbiome	6 (100%)	16 (64%)	7 (58%)	0.184^^^

^^^*p* value calculated using Monte Carlo to stimulate *p* value with Fisher’s exact test, based on 2000 iterations

^χ^*p* value calculated using the Kruskal-Wallis test

^a^Self-reported vaginal symptoms including pain, discomfort, itchiness, discharge, bleeding

### Antigen-presenting cells differ based on vaginal inflammation status

**Fig. 2:**
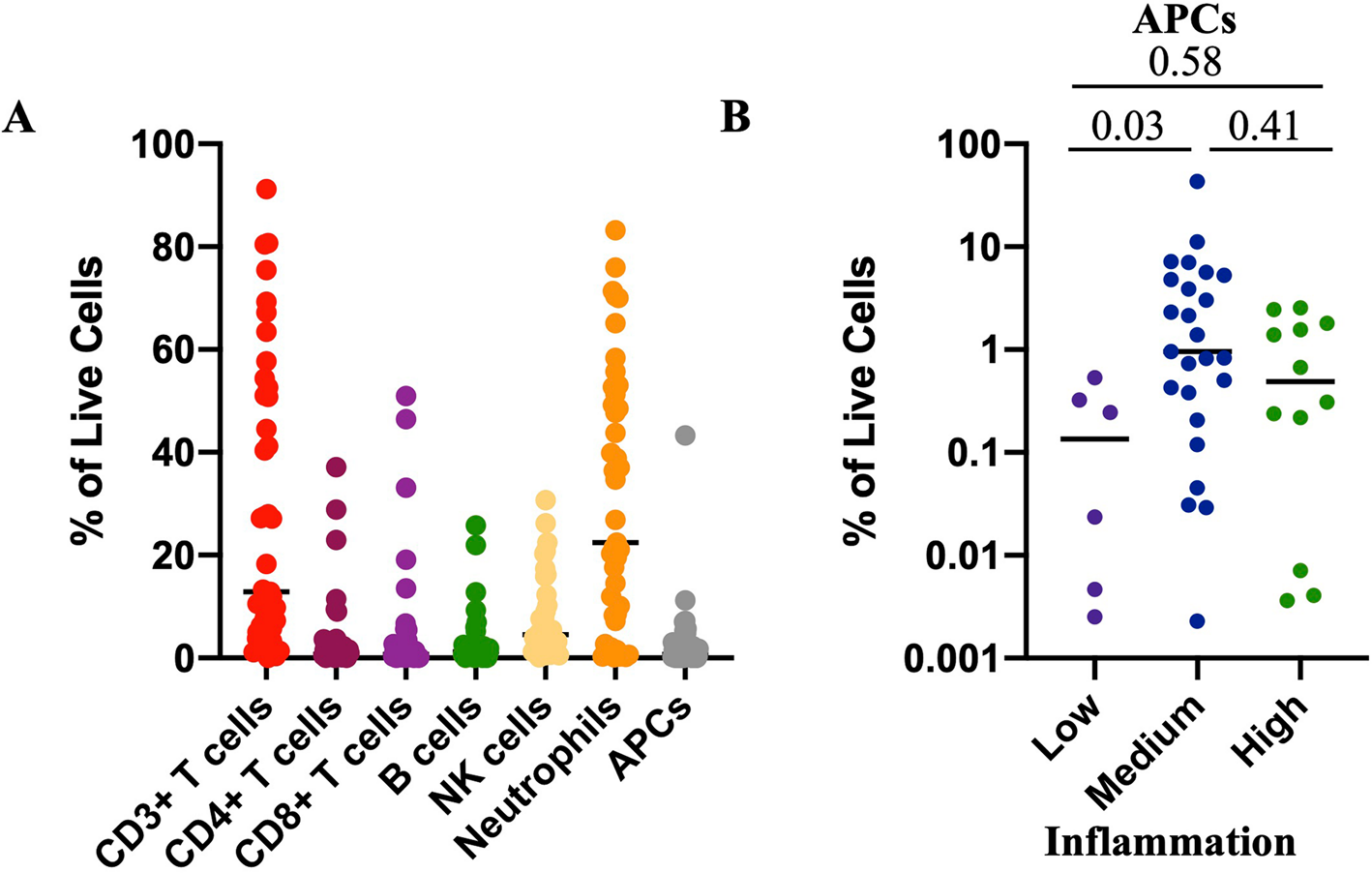
Flow cytometry analysis associates higher cervical antigen-presenting cells with increased genital inflammation. **A** Cervical leukocytes were immunophenotyped by flow cytometry to identify immune cell lineages. **B** APCs identified from cervical cytobrushes, stratified by inflammation level defined by the presence of inflammatory cytokines in CVL. Adjusted *p* values are reported and were calculated using the Kruskal-Wallis test with Dunn’s correction for multiple comparisons. Data is presented as a percentage of total live cells identified by flow cytometry with median identified. *N*=43

As vaginal inflammation has been associated with increased immune cell infiltration, we used multiparameter flow cytometry to phenotype endocervical immune cells collected from study participants to detect immune cell lineages (CD4+ T cells, CD8+ T cells, CD19+ B cells, CD56+ Natural Killer (NK) cells, CD14+ antigen-presenting cells (APCs), and CD15+CD16+CD49d- neutrophils) (Fig. 2A, Supplemental Figure 1). Neutrophils (mean 30.3% range 0.13–83.17%) were the most abundant immune cell present, followed by CD3+ T cells (mean 28% range 0.0–91.2%) and NK cells (mean 7.6% range 0.14–30.68%). When we examined immune cell differences based on inflammation status, APCs were the only cell type that differed by inflammation (Kruskal-Wallis *p*=0.025, *H* statistic=7.39, Degrees of freedom=2) (Fig. 2B), with significantly higher levels of APCs in participants in the medium compared to low inflammation groups (p.adj=0.03). No immune cell subsets differed by *Lactobacillus* dominance of the microbiome.

### Vaginal inflammation is associated with proteome alterations related to increased innate immunity and decreased epithelial barrier integrity

**Fig. 3:**
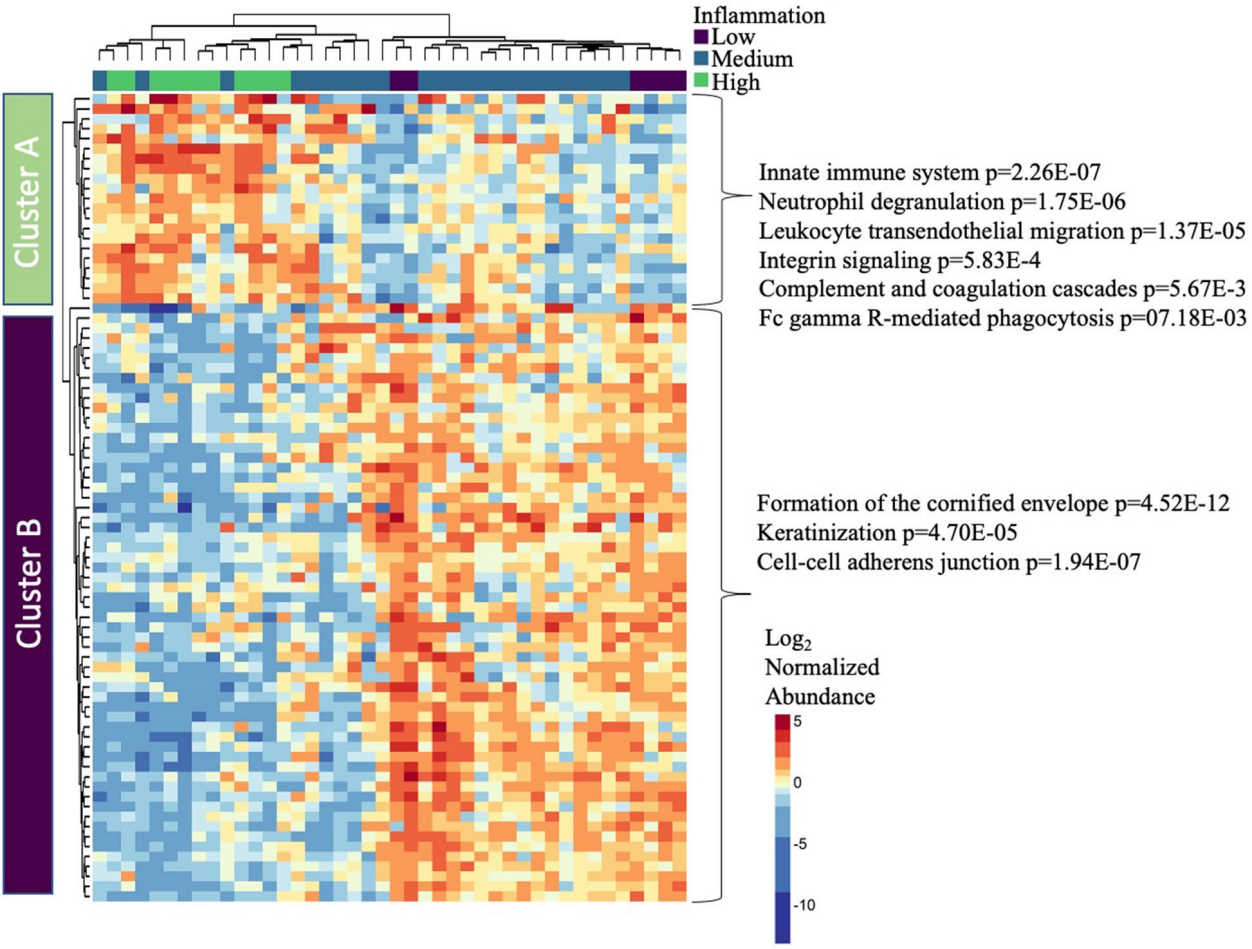
Cervicovaginal inflammation associates with neutrophil and epithelial barrier proteome changes. Mass spectrometry analysis of cervicovaginal fluid identified alterations to the mucosal proteome with inflammation groups. Proteins that were differentially abundant (adj. *p*<0.05) underwent unsupervised hierarchical clustering, and inflammation status based on levels of inflammatory cytokines was overlaid. Two clusters of proteins were identified. Proteins from each cluster underwent pathway analysis using ConsensusPathDB-human and significant pathways are shown on the right. One participant (high inflammation group) did not have a sample available for mass spectrometry analysis. Differential protein expression analysis was performed using the Kruskal-Wallis test with Benjamini-Hochberg correction for multiple comparisons. *N*=42

Using mass spectrometry-based proteomics there were 925 human proteins identified within CVL. There were 275 (29.8%) proteins that were significantly different by inflammation group (*p*<0.05), with 81 (8.8%) passing correction for multiple comparisons (p.adj<0.05). These 81 proteins clustered by inflammation group using unsupervised hierarchical clustering (chi-square *p*<0.0001) into “high inflammation” and “medium/low inflammation” branches, and two main protein clusters were identified (Fig. 3). Cluster A contained 21 proteins, which are increased in all (11/11) participants with high inflammation and 36% (9/25) of the medium inflammation participants. Proteins in cluster A were associated with biological pathways related to innate immune system (*p*=2.26E−07), including neutrophil degranulation (*p*=1.75E−06), integrin signaling (*p*=5.83E−04), and leukocyte migration (*p*=1.37E−05), indicating there are higher proteome signals of inflammation in participants with higher levels of cytokine-defined inflammation. Cluster B contained 60 proteins, which tend to be lower in participants with high inflammation. Proteins within Cluster B are associated with tissue integrity pathways, including cell-cell adherens junction (*p*=1.94E−07), cornified envelope (*p*=4.52E−12), and keratinization (*p*=4.70E−05), indicating decreased epithelial barrier integrity or increased barrier disruption with high inflammation. The “high inflammation” branch, which had increases in proteome signatures related to innate immunity, contained participants that had significantly higher levels of neutrophils (*p*=0.0247), which supports the findings of the pathway analysis. While there were 134 host proteins that differed between *Lactobacillus* dominant (LD) and non-*Lactobacillus* dominant (nLD) groups, none passed correction for multiple comparisons.

### *L. crispatus* is higher in participants with low genital inflammation

**Fig. 4:**
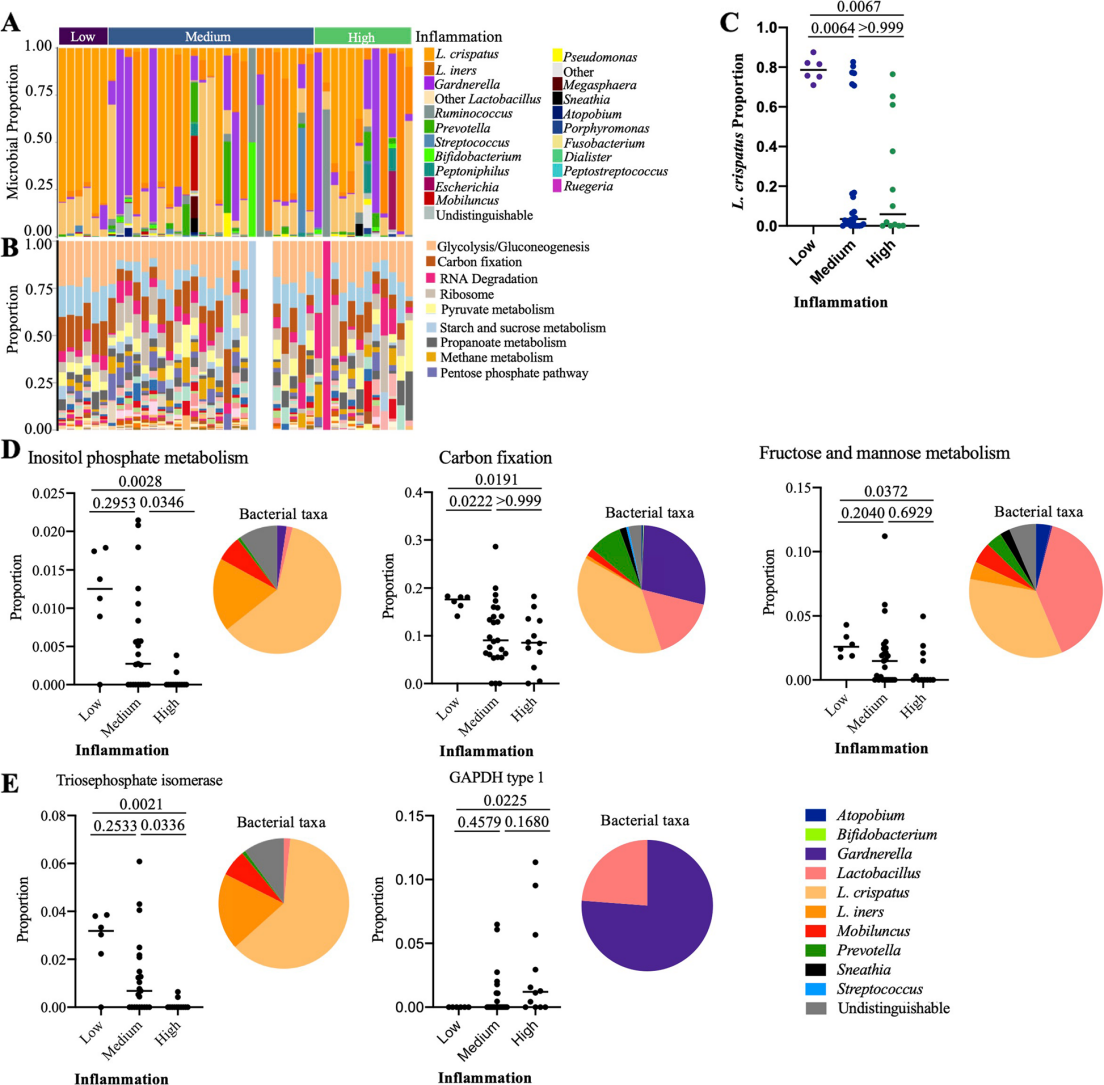
Cervicovaginal microbiome composition and functional differences between women with high and low genital inflammation. Proportion plots of **A** taxa and **B** ko-level bacterial functions identified in cervicovaginal lavage by mass spectrometry. Participants are grouped by inflammation status. Two participants in the medium inflammation group did not have bacterial functions mapped to the identified bacterial proteins. **C** Microbial proportion of *L. crispatus* grouped by inflammation status with median identified. **D** Proportion of ko-level bacterial functions by inflammation status. Pie charts show the distribution of bacterial taxa for each function. **E** Bacterial proteins that differed by inflammation status. Pie charts show the distribution of bacterial taxa for each protein. Adjusted *p* values are reported and were calculated using the Kruskal-Wallis test with Dunn’s correction for multiple comparisons. *N*=43

As it has been observed that the vaginal microbiome can have a significant impact on inflammation, we used mass spectrometry-based metaproteomics to detect bacterial proteins present in CVL. 1758 unique bacterial proteins from 19 genera were identified by mass spectrometry (Fig. 4). *L. crispatus*, *L. iners*, *Gardnerella*, and other *Lactobacillus* species were the most abundant bacteria detected. When participants were classified based on the abundance of *Lactobacillus* detected, with women having >50% of their bacterial proteins from *Lactobacillus* considered *Lactobacillus* dominant (LD), we found that all participants with low inflammation (6/6, 100%), 16 from the medium inflammation group (64%), and 7 from the high inflammation group (58%) were LD, although this was not statistically different (*p*=0.184). Relative abundance of *L. crispatus* was significantly different based on the inflammation group (Kruskal-Wallis *p*=0.004, H statistic=10.83, Degrees of freedom=2), and was higher in the low inflammation group compared to both the medium (p.adj=0.0064) and high (p.adj=0.0067) groups (Fig. 4C). *Ruminococcus* was also statistically different between inflammation groups (Kruskal-Wallis *p*=0.019 H statistic=7.93, Degrees of freedom=2), with a significant increase in medium compared to low inflammation (p.adj=0.020). However, this bacterium was in low abundance and the differences appeared to be driven by 3 participants that had higher levels of this bacteria. No other bacteria were significantly different between inflammation groups.

KEGG gene ontology was used to map the bacterial proteins to functional pathways, with 971 (55%) bacterial proteins mapped to 33 ko-level pathways. The most abundant functions were glycolysis/gluconeogenesis, carbon fixation, and RNA degradation. There were 5 ko-level pathways that were significantly different by inflammation group, inositol phosphate metabolism (*p*=0.002, p.adj=0.074), two-component system (*p*=0.007, p.adj=0.119), carbon fixation (*p*=0.014, p.adj=0.145), amino sugar and nucleotide sugar metabolism (*p*=0.018, p.adj=0.145), and fructose and mannose metabolism (*p*=0.041, p.adj=0.233), although none passed correction for multiple comparisons (Fig. 4D). There were 25 bacterial proteins identified that were significantly different by inflammation status, but none passed correction for multiple comparisons. Of these proteins, eight (glutamine synthetase, glyceraldehyde-3-phosphate dehydrogenase, glyceraldehyde-3-phosphate dehydrogenase type 1, phosphate binding protein, phosphoenolpyruvate carboxykinase (ATP), phosphoglucosamine mutase, triosephosphate isomerase, and uncharacterized protein) were present in the ko-level pathways that differed by inflammation group. Triosephosphate isomerase (*p*=0.0018), which was identified in the fructose and mannose metabolism, inositol phosphate metabolism, and carbon fixation pathways, was significantly lower in the high inflammation group compared to both the medium (p.adj=0.0336) and low (p.adj=0.0021) inflammation groups. Glyceraldehyde-3-phosphate dehydrogenase type 1 (*p*=0.0212), identified in the carbon fixation pathway, was the only protein that was higher in the high inflammation group compared to the low inflammation group (p.adj=0.0225) (Fig. 4E), suggesting a change in bacterial metabolism may be associated with increasing inflammation.

### Vaginal xanthine is significantly higher in women with high levels of vaginal inflammation

**Fig. 5:**
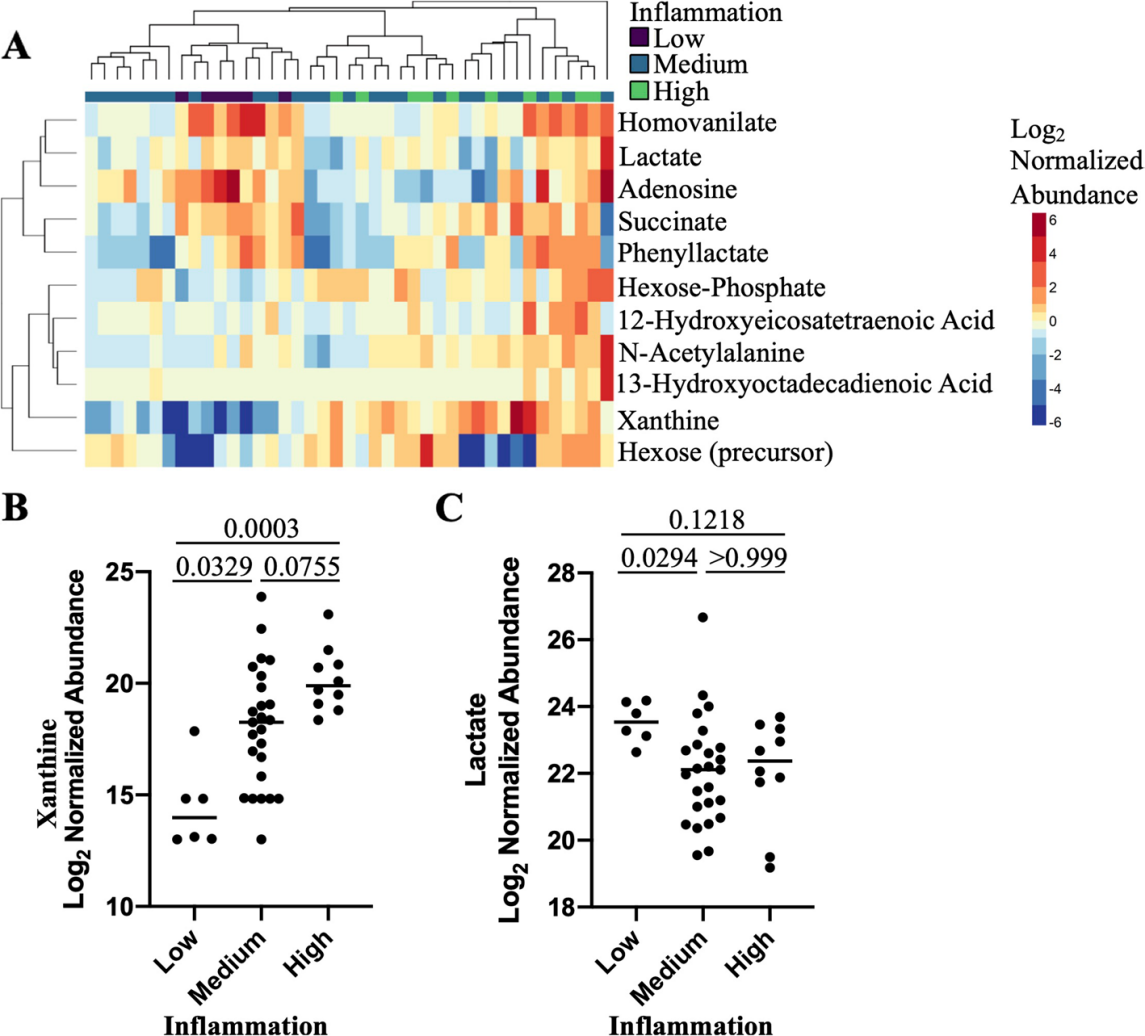
Cervicovaginal metabolome differences between women with high and low inflammation. **A** Hierarchical clustering of differentially abundant (*p*<0.05) metabolites based on inflammation group. **B** Log2 normalized abundance of xanthine identified in CVL samples by inflammation group with median indicated. **C** Log2 normalized abundance of lactate identified in CVL samples by inflammation group with median indicated. Adjusted *p* values are reported and were calculated using the Kruskal-Wallis test with Dunn’s correction for multiple comparisons. Two participants from the high inflammation group did not have samples available for metabolomics analysis. *N*=41

To investigate potential communications between the host and the microbiome, we used targeted metabolomics to identify metabolites in CVL. A total of 82 metabolites were identified including amino acids, short chain fatty acids, organic acids, fatty acids, carbohydrates, nucleic acids, and amines. Eleven (13.4%) metabolites were significantly different based on the inflammation group (*p*<0.05, Kruskal-Wallis test; Fig. 5A), including fatty acids (4 metabolites), organic acids (2 metabolites), nucleic acids (2 metabolites), sugars (2 metabolites), and amino acids (1 metabolite). The purine nucleic acid xanthine was the top metabolite associated with inflammation (Kruskal-Wallis *p*=6.00E−4, BH p.adj=0.046, H statistic=14.99, Degrees of freedom=2), and is higher in both the medium (Dunn’s p.adj=0.033) and high (Dunn’s p.adj=0.0003) inflammation groups compared to women with low inflammation, and increased in the high group compared to the medium group (p.adj=0.076), although this did not pass statistical significance (Fig. 5B). Other metabolites increased in participants with high inflammation included hexose-phosphate (*p*=0.0115, p.adj=0.21), hexose (*p*=0.0274, p.adj=0.28), N-acetyl alanine (*p*=0.0471, p.adj=0.33), 12-hydroxyeicosatetraenoic acid (*p*=0.0125, padj=0.21), and 13-hydroxyoctadecadienoic acid (*p*=0.0331, p.adj=0.28), although none passed correction for multiple comparison. Metabolites that were higher in participants with low inflammation included homovanilate (*p*=0.0125, p.adj=0.21), lactate (*p*=0.034, p.adj=0.28), adenosine (*p*=0.0075, p.adj=0.21), and succinate (*p*=0.0207, p.adj=0.28), although none passed correction for multiple comparisons (Fig. 5C). Interestingly, phenyllactate (*p*=0.0233, p.adj=0.28) was lowest in the participants with medium inflammation. Unsupervised hierarchical clustering indicated these metabolites tended to cluster by inflammation group (chi-square *p*=0.101) and did not cluster by *Lactobacillus* dominance of the microbiome (Fisher’s exact *p*=0.469). There were 26 (31.7%) metabolites that differed based on microbiome status, with 14 (cytosine p.adj=0.033; inosine p.adj=0.033; glycine p.adj=0.033; lactate p.adj=0.033; methionine sulfoxide p.adj=0.033; tyrosine p=0.034; adenosine p.adj=0.034; serine p.adj=0.036; uridine p.adj=0.039; tryptophan p.adj=0.039; hexose p.adj=0.047; glutamate p.adj=0.047; leucine-isoleucine p.adj=0.047; xanthine p.adj=0.047) passing correction for multiple comparisons. Of these 14 metabolites, 3 overlapped with the inflammation metabolomic signature (xanthine, adenosine, and lactate) suggesting these metabolites may be important for microbiome-inflammation interactions (Supplemental Table 1).

### Bayesian network analysis identifies molecular relationships with inflammation

**Fig. 6:**
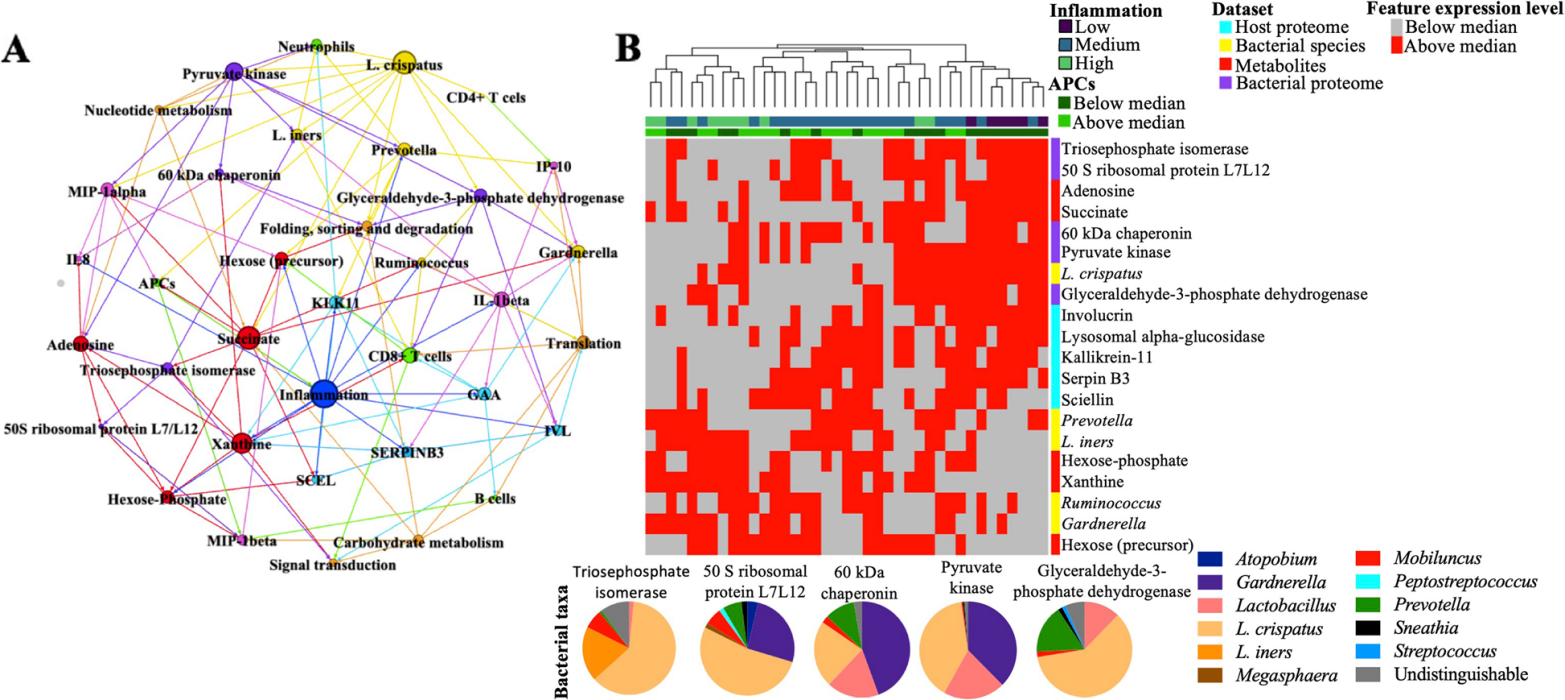
Bayesian network analysis of cervicovaginal inflammation. Major features are colored by dataset. Node size is representative of the number of edges per node, with larger nodes having more edges (**A**). Heatmap of the top five features selected by Random Forest based on Gini importance from host proteome, microbiome, bacterial protein, and metabolome datasets (**B**). Each feature was assigned to above or below median level and used for unsupervised hierarchical clustering. Inflammation status and levels of APCs are overlaid. Samples significantly cluster by inflammation status (chi-square *p*=5.34 × 10^−5^). Pie charts show the distribution of bacterial taxa for each protein included in the heatmap

To better understand the potential relationships between cervicovaginal inflammation and the microenvironment, we employed an integrated Bayesian network method. The cytokine, immune cell, proteome, metaproteome (species, functional pathway (b level), protein), and metabolome datasets were pre-filtered using random forest models, and the top 5 features were selected from each dataset, with the inflammation variable also included, leading to a total of 36 features. The final model identified 119 edges between 36 nodes (Fig. 6A). Inflammation, *L. crispatus*, and succinate were major nodes identified. Inflammation status was related to the neutrophil chemotactic factor IL-8, pro-inflammatory cytokine IL-1β, and to APCs. Primary interactions with inflammation status included links to epidermal integrity proteins (SCEL and IVL), as well as metabolites, including xanthine and carbohydrates hexose and hexose-phosphate. Primary microbiome interactions included anaerobic taxa *Prevotella* and *Ruminococcus*. A second major node identified was *L. crispatus*, which was linked to *L. iners* and *Gardnerella*. *L. crispatus* was also directly linked to the inflammatory cytokine MIP-1α, and the immune cells APCs, neutrophils, and CD4+ T cells. Succinate is a metabolite that has been associated with BV, and was linked to both *L. crispatus* and *Gardnerella* in this network. While succinate was not linked directly to inflammation, it was linked to MIP-1α and APCs. Succinate was also linked to bacterial carbohydrate metabolism, carbohydrate metabolites, and the human protein SCEL, which is part of the cornified envelope. When metabolites, microbiome features, and host proteins were classified as above or below median levels and underwent unsupervised hierarchical clustering, we found that samples cluster by inflammation (chi-square *p*=5.34 × 10^−5^) and level of APCs (FET *p*=0.031) (Fig. 6B), indicating a potential shift in metabolic pathways with increases in inflammation, which was also reflected in shifts in bacterial metabolism based on the bacterial proteome. Overall, this network highlights several novel interactions between APCs, metabolites, and bacterial functions that may be important for mediating cervicovaginal inflammation.

## Discussion

Sustained cervicovaginal inflammation can have a detrimental impact on the cervicovaginal mucosa, leading to epithelial barrier damage and increased immune cell infiltration or activation, resulting in adverse health outcomes. In this study of women living in Canada, we show that APCs are significantly higher with vaginal inflammation and linked to the microbiome, the metabolite succinate, loss of functional bacterial pathways, epithelial barrier disruption, and neutrophil-related activation pathways. In addition, we identified the metabolite xanthine as another key feature of increased inflammation. Overall, this study shows a complex interplay between shifts in bacterial metabolism to increased pro-inflammatory cytokines and changes to innate immunity in the female genital tract.

APCs are important sensors of the local microenvironment, linking innate and adaptive immunity. Work by others has found that APC frequency is not significantly different based on microbiome group, but that there are pronounced transcriptional differences in cervical APCs from women with *Lactobacillus* compared to non-*Lactobacillus* microbiomes [27, 68]. APCs from women with non-*Lactobacillus* microbiomes exhibited upregulation of pro-inflammatory mediators including TNFα signaling pathways and increased expression of pro-inflammatory cytokine genes compared to *Lactobacillus* dominant women [27, 68]. These APCs were also more activated and mature, which could contribute to subsequent T cell priming and control of effector functions [27]. In this study, we did not observe any difference in APC levels based on *Lactobacillus* dominance but there was a relationship between APCs and lower *L. crispatus*. There were no significant correlations between non-*Lactobacillus* microbiota and APCs, which may be due to the lower levels of these bacteria in this cohort. APCs were also linked to the metabolite succinate, which was identified as a major molecular feature of inflammation. Succinate has previously been linked to BV [52] which agrees with our findings that succinate associates with *L. crispatus*, *Gardnerella*, and *Ruminococcus*. Succinate is a pro-inflammatory metabolite and can augment TLR-induced production of TNF and IL-1β and trigger chemotaxis and activation of APCs [69, 70]. In addition, succinic acid can significantly increase HIV expression in infected macrophages and increase IL-8 production by virus-infected cells [71]. Thus, succinate could be a metabolite linking the vaginal microbiome to APCs and subsequent inflammation in the cervicovaginal mucosa. As subsets of APCs can be infected by HIV and have been associated with both parturition [72–76] and progression of solid tumors, including cervical cancer [16, 77–80], this interaction between inflammation, the microbiome, and metabolites could be an important component of reproductive health.

Proteomics analysis confirmed the inflammation grouping of participants, with those having high inflammation having increases in pathways related to immune cell infiltration, phagocytosis, and neutrophil degranulation. Interestingly, APCs were the only immune cell increased in participants with high inflammation, suggesting that higher levels of inflammation may represent functional or phenotypic changes in immune cell subsets that may not be reflected by immune cell lineage analysis. Follow-up studies are further characterizing cervical immune cell populations to investigate functional differences that may exist. Participants with high levels of inflammation also had decreases in pathways related to epithelial barrier integrity, which supports a relationship between increased inflammatory cytokines and epithelial barrier damage. Indeed, we have previously shown that elevated levels of cytokines were associated with mucosal proteome signatures of decreased barrier proteins [4]. Inflammation was directly linked to the barrier proteins sciellin (SCEL), serpin B3 (SERPINB3), and involucrin (IVL). While there were no direct links identified between these proteins and the microbiome in our analysis, they did link to bacterial functions such as carbohydrate metabolism and signal transduction, and to metabolites including succinate, xanthine, and hexose-phosphate, suggesting an indirect link between barrier damage and the microbiome via bacterial metabolism.

The microbiome can be a major contributor to mucosal inflammation [6]. *L. crispatus* was significantly higher in participants with low inflammation compared to both the medium and high inflammation groups, which supports the findings of other studies [1, 10, 14–16]. Inflammation was directly linked to *Prevotella* and *Ruminococcus*. *Prevotella* has a recognized role in BV and is linked to inflammation and increased cytokine production [81] and has been linked to increased inflammation in other studies [1, 27, 28, 68]. In mouse models, vaginal introduction of *Prevotella* increased IL-6 and IL-8 production and recruited and activated CD4+ T cells in the genital mucosa compared to *L. crispatus* inoculation [21]*. Prevotella* was linked directly to neutrophils and IP-10, suggesting these are potential mediators of *Prevotella*-associated inflammation. *Ruminococcus* has been identified in the vaginal microbiota in other studies [64, 82], but its role in inflammation has not been studied. *Ruminococcus* was linked directly to IL-1β and CD8+ T cells, although directionality of this is not known, so it is difficult to determine the effect of *Ruminococcus* on inflammation. In addition, *Ruminococcus* was a low-abundance bacterial species identified, suggesting larger studies are needed to better understand its potential role in the cervicovaginal microenvironment.

The functional metaproteomic data indicated changes in bacterial metabolism associated with inflammation. Interestingly, lactate and bacterial enzymes such as lactate dehydrogenase, which are typically associated with low inflammation [28, 56, 83, 84], were not among the top features selected, whereas triosephosphate isomerase, detected primarily from *L. crispatus*, was selected. This suggests that enzymes involved upstream of homolactic fermentation may be better indicators or more important contributors to metabolic stability that are needed to maintain low levels of inflammation. This information may be helpful for the design of microbiome-based therapeutic interventions that are targeting the vaginal microbiome and selection parameters for *L. crispatus* strains for inclusion in these interventions. Inositol phosphate metabolism was also significantly decreased in participants with high inflammation. Microbiota-derived inositol phosphate has been found to regulate histone deacetylase (HDAC) activity in the gut, with commensal bacteria stimulating HDAC activity through inositol triphosphate production and promoting epithelial repair in a mouse model of colitis [85, 86]. In support of this, we found that high inflammation associated with decreased human pathways of epithelial barrier integrity, including significant decreases in the keratinocyte-associated proteins IVL and SCEL.

Xanthine was identified as a major metabolite underlying increased inflammation. Xanthine is a purine base that is found in most human tissues and fluids and its role in inflammation is not well studied, although it has been demonstrated to be increased in amniotic fluid following LPS exposure in mouse models and increased in women that had a spontaneous first-trimester miscarriage [87, 88]. Xanthine is a degradation product of inosine, which has been studied in the context of both the gut microbiome and immunity [89, 90]. Increases in microbiome-derived inosine can promote antitumor immunity, which is dependent on the activation of CD4+ Th1 cells [89]. While increased levels of xanthine were also observed in this study, mechanistic experiments were only performed with inosine [89]. In addition, effector CD8+ T cells can utilize inosine to support cell growth and function in the absence of glucose in vitro [90]. While inosine was detected by metabolomics in this study, it was not associated with inflammation status. Future studies should investigate the impact of both xanthine and inosine on cervicovaginal T cell function.

A major strength of this study is the in-depth multi-‘omics analysis that was performed, including cytokines, multiparameter flow cytometry, metaproteomics, and metabolomics on paired samples, allowing us to undertake more complex analysis such as Bayesian network to investigate potential interactions. While the Bayesian network analysis is descriptive and allows the identification of potential relationships for future mechanistic studies, several of the interactions identified support published data. In addition, this study investigated cervicovaginal inflammation in participants that would be considered healthy, illustrating potential factors that could contribute to an inflammatory microenvironment in the absence of disease. Limitations of this study include the small sample size and lack of microbiome diversity, with only 8 participants (19%) having a clinical diagnosis of BV, which is a major modulator of the cervicovaginal microenvironment. In addition, CD45, as a marker of hematopoietic-derived immune cells, was not included in the flow cytometry panel. While this precludes CD45 vs SSC gating, which could impact the identification of leukocyte subsets within samples, all immune cell subtypes were identified using lineage-specific markers. Finally, data on the menstrual phase and menopause was not collected. As there was no upper age limit for recruitment into this study, the average participant age was high, and it is likely that several participants had reached menopause. While it is important to study the impacts of age or menopause on the cervicovaginal microenvironment, the hormonal and microbiome changes that occur over the menstrual cycle and with menopause may confound the findings.

In conclusion, this study identifies interactions between microbial metabolism and the cervicovaginal microenvironment that could modulate inflammation and host immunity. These data may have implications for susceptibility to infections in the female genital tract and other reproductive health outcomes. Further studies are needed to explore the interactions between bacterial metabolism, mucosal immunity, APCs, and genital inflammation.

## Supplementary Information

**Additional file 1: Supplemental Table 1.** Metabolites that differ by inflammation status or *Lactobacillus* dominance.

**Additional file 2: Supplemental Figure 1.** Representative overview of gating strategy. CMCs were dislodged from cytobrushes and stained with an optimized antibody cocktail to identify immune cell lineages. Data was acquired on an LSRII (BD Biosciences) and analyzed using FlowJo. Forward and side scatter were used to identify cells, and then singlets were gated. A fixable viability dye was used to identify live cells and then cells were gated based on expression of CD3. CD3+ cells were gated to determine expression of CD4 and CD8. CD3- cells were examined for expression of CD19 and CD56. CD19-CD56- cells were examined for expression of CD14. CD14- cells were examined for expression of CD15 and CD16, then CD49d expression on CD15+CD16+ was examined. PBMCs were used for instrument set up and gating including unstained PBMCs, single colour controls, and FMOs.

**Additional file 3.** Datasets. 

**Additional file 4. **Proteomics results_batch 1.

**Additional file 5.** Proteomics results_batch 2.

**Additional file 6.** Metaproteome results.

## Data Availability

Data sets including cytokine measurements, immune cell levels, metaproteome, and metabolome have been included as a supplemental file.
