# Supplementary material for: Increased genital mucosal cytokines in Canadian women associate with higher antigen-presenting cells, inflammatory metabolites, epithelial barrier disruption, and the depletion of L. crispatus
Source: Microbiome. 2023 Jul 25;11:159. doi: 10.1186/s40168-023-01594-y (PMC10367425; doi:10.1186/s40168-023-01594-y)
Supplement: Supplementary file 2 — Additional file 1: Supplemental Table 1. Metabolites that differ by inflammation status or Lactobacillus dominance. [file 40168_2023_1594_MOESM1_ESM.docx]

**Supplemental Table 1:** Metabolites that differ by inflammation status or *Lactobacillus* dominance

| **Metabolite** | **Analysis_group** | **test.used** | **P.value** | **adj.method** | **adj.Pvalue** |
| --- | --- | --- | --- | --- | --- |
| Xanthine | Inflammation | Kruscal | 0.00055573 | BH | 0.04612555 |
| Adenosine | Inflammation | Kruscal | 0.00752466 | BH | 0.20796727 |
| Hexose-Phosphate | Inflammation | Kruscal | 0.01153706 | BH | 0.20796727 |
| Homovanilate | Inflammation | Kruscal | 0.01248657 | BH | 0.20796727 |
| 12-Hydroxyeicosatetraenoic Acid | Inflammation | Kruscal | 0.01252815 | BH | 0.20796727 |
| Succinate | Inflammation | Kruscal | 0.02066186 | BH | 0.27576098 |
| Phenyllactate | Inflammation | Kruscal | 0.02325695 | BH | 0.27576098 |
| Hexose-2 (precursor) | Inflammation | Kruscal | 0.02743952 | BH | 0.28199715 |
| 13-Hydroxyoctadecadienoic Acid | Inflammation | Kruscal | 0.03311114 | BH | 0.28199715 |
| Lactate | Inflammation | Kruscal | 0.03397556 | BH | 0.28199715 |
| N-Acetyl Alanine | Inflammation | Kruscal | 0.04708623 | BH | 0.33118385 |
| Cytosine | LD | Wilcoxon | 0.00095932 | BH | 0.03250526 |
| Inosine | LD | Wilcoxon | 0.00131126 | BH | 0.03250526 |
| Glycine(precursor) | LD | Wilcoxon | 0.00135007 | BH | 0.03250526 |
| Lactate | LD | Wilcoxon | 0.00168537 | BH | 0.03250526 |
| Methionine Sulfoxide | LD | Wilcoxon | 0.00195815 | BH | 0.03250526 |
| Tyrosine | LD | Wilcoxon | 0.00258483 | BH | 0.0339976 |
| Adenosine | LD | Wilcoxon | 0.00286727 | BH | 0.0339976 |
| Serine | LD | Wilcoxon | 0.00351493 | BH | 0.03646738 |
| Uridine | LD | Wilcoxon | 0.00444523 | BH | 0.03923866 |
| Tryptophan | LD | Wilcoxon | 0.00472755 | BH | 0.03923866 |
| Hexose-1 | LD | Wilcoxon | 0.00681074 | BH | 0.04747649 |
| Glutamate | LD | Wilcoxon | 0.00690503 | BH | 0.04747649 |
| Leucine-Isoleucine | LD | Wilcoxon | 0.00756959 | BH | 0.04747649 |
| Xanthine | LD | Wilcoxon | 0.00800808 | BH | 0.04747649 |
| Phenylalanine | LD | Wilcoxon | 0.0108135 | BH | 0.05983468 |
| Urea | LD | Wilcoxon | 0.01280934 | BH | 0.06521872 |
| Aspartate | LD | Wilcoxon | 0.01397387 | BH | 0.06521872 |
| Asparagine | LD | Wilcoxon | 0.01414382 | BH | 0.06521872 |
| Pyroglutamate-3 | LD | Wilcoxon | 0.01649416 | BH | 0.06711381 |
| Lysine | LD | Wilcoxon | 0.01649416 | BH | 0.06711381 |
| Deoxyinosine | LD | Wilcoxon | 0.01722027 | BH | 0.06711381 |
| Methionine | LD | Wilcoxon | 0.0185978 | BH | 0.06711381 |
| Valine | LD | Wilcoxon | 0.0245393 | BH | 0.08486508 |
| Threonine | LD | Wilcoxon | 0.02649212 | BH | 0.08795384 |
| Homovanilate | LD | Wilcoxon | 0.03004419 | BH | 0.0959103 |
| Arginine | LD | Wilcoxon | 0.032084 | BH | 0.09862859 |
