# Supplementary figures and images for: Increased genital mucosal cytokines in Canadian women associate with higher antigen-presenting cells, inflammatory metabolites, epithelial barrier disruption, and the depletion of L. crispatus
Source: Microbiome. 2023 Jul 25;11:159. doi: 10.1186/s40168-023-01594-y (PMC10367425; doi:10.1186/s40168-023-01594-y)

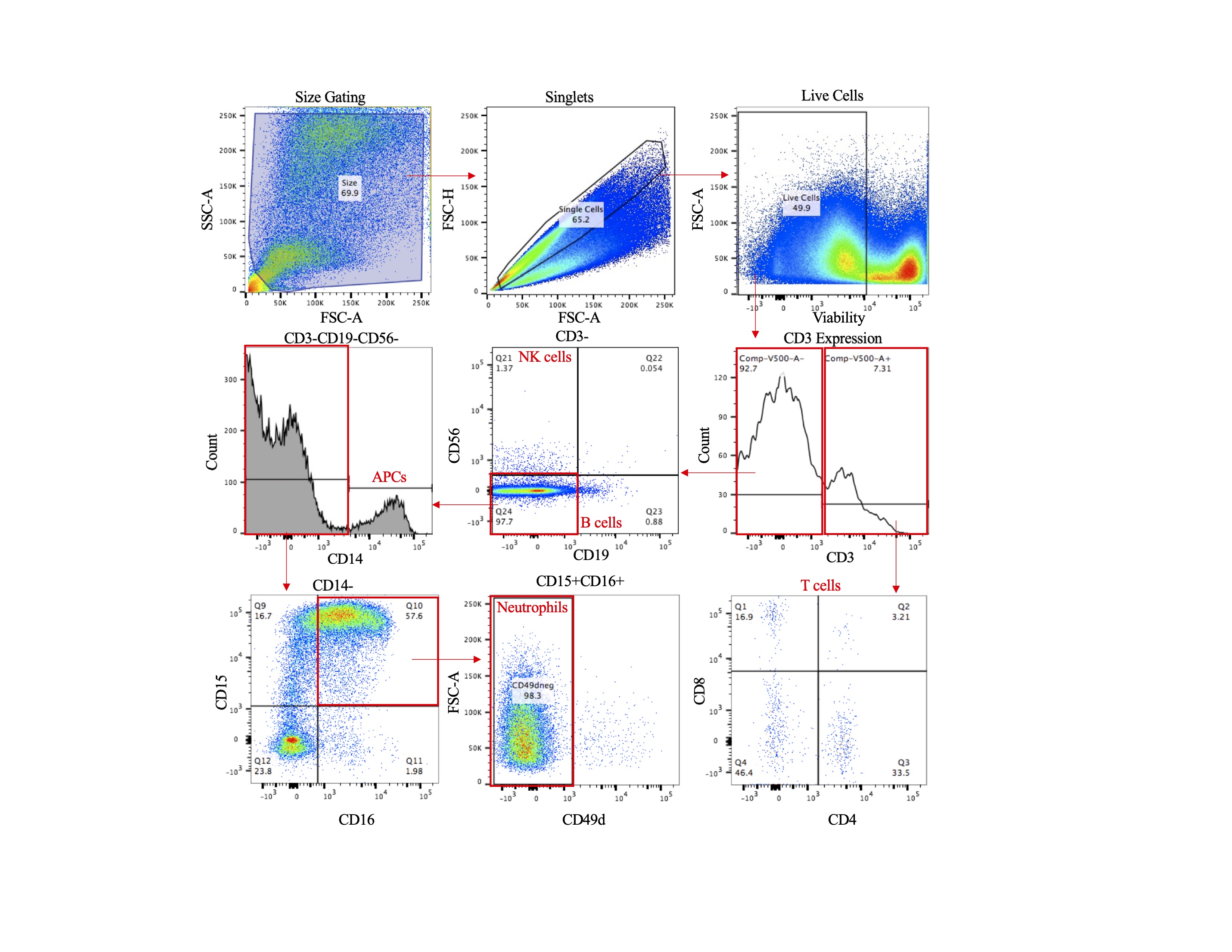

Supplement: Supplementary file 3 — Additional file 2: Supplemental Figure 1. Representative overview of gating strategy. CMCs were dislodged from cytobrushes and stained with an optimized antibody cocktail to identify immune cell lineages. Data was acquired on an LSRII (BD Biosciences) and analyzed using FlowJo. Forward and side scatter were used to identify cells, and then singlets were gated. A fixable viability dye was used to identify live cells and then cells were gated based on expression of CD3. CD3+ cells were gated to determine expression of CD4 and CD8. CD3- cells were examined for expression of CD19 and CD56. CD19-CD56- cells were examined for expression of CD14. CD14- cells were examined for expression of CD15 and CD16, then CD49d expression on CD15+CD16+ was examined. PBMCs were used for instrument set up and gating including unstained PBMCs, single colour controls, and FMOs. [file 40168_2023_1594_MOESM2_ESM.jpg]
